# Expression of SIRT1 and DBC1 in Developing and Adult Retinas

**DOI:** 10.1155/2012/908183

**Published:** 2012-08-30

**Authors:** Shawn C. Maloney, Emilia Antecka, Alexandre N. Odashiro, Bruno F. Fernandes, Madeline Doyle, Li-Anne Lim, Yousef Katib, Miguel N. Burnier

**Affiliations:** Henry C. Witelson Ocular Pathology Laboratory, McGill University, Montreal, QC, Canada H3A 2B4

## Abstract

Sirtuin 1 (SIRT1) is a deacetylase that can regulate various biological processes via repression of transcription. Its activity has been linked to the differentiation of neural progenitor cells, although little is known about its function during retinal development. The study described herein was undertaken to evaluate the expression of SIRT1 and its innate inhibitor, DBC1, in retinal tissues and progenitor cells. We found both SIRT1 and DBC1 to be widely expressed in mouse and human retinas, with subtle differences in subcellular distribution of each protein. We further demonstrate that nuclear-localized SIRT1 is only seen in human-derived retinal progenitor cells and not in adult retinas, suggesting that this nuclear localization may be important in retinal development. Moreover, we observed cytoplasmic DBC1 in a subset of progenitor cells as well as in mature ganglion cells, indicating that the progenitor cell subset, which was comprised predominantly of small cells, may represent a population of ganglion cell precursors. Collectively, the data presented in this study provide support for SIRT1 and DBC1 as regulators of retinal development and normal retinal physiology.

## 1. Introduction

Sirtuin 1 (*SIRT1*) is the mammalian ortholog of the yeast *Sir2* gene, which has been shown to play a role in extending replicative lifespan via repression of genomic instability [1, 2]. Despite its classification as a histone deacetylase, SIRT1 has a number of nonhistone substrates, including p53 [3], NF-*κ*B [4], PGC-1*α* [5], and FOXO [6]. Extensive research has shown that SIRT1 plays a critical role in various biological processes, including cellular senescence [7], glucose metabolism [5], antiapoptosis [3], neuronal protection [8], insulin secretion [9], angiogenesis [10], and neural development [11].

Research has shown that SIRT1 is highly expressed during embryogenesis [12]. Studies of *Sirt1* knockout mice have revealed a number of phenotypic differences compared to wildtype mice, including cardiovascular defects and abnormal retinal architecture [11]. Given that targets of SIRT1 include master transcription factors and cell cycle regulators, it is not surprising that *Sirt1* abrogation or deletion causes severe phenotypic consequences. Despite publications that support a role for SIRT1 in retinal development, at least one study has reported evidence to the contrary [13]. Thus, the potential role of SIRT1 in retinal development is currently a matter of debate.

Hisahara and colleagues recently demonstrated that SIRT1 could influence the differentiation of neural progenitor cells (NPCs) [14]. SIRT1 was found to shuttle from the cytoplasm to cell nuclei, following addition of differentiation-inducing stimuli, and back to the cytoplasm within six hours. Inhibition of SIRT1 reduced the ratio of neural-to-glial cells derived following differentiation, whereas overexpression of *SIRT1* led to a higher yield of mature neural cells. As SIRT1 contains both nuclear localization and nuclear export signals, its subcellular distribution may be governed by these signals which could be affected by cell type, stress, and molecular interactions [15].

While studies have been undertaken to elucidate the roles of SIRT1 in the CNS, little is known about its role in the retina. A deeper understanding of whether or not SIRT1 affects retinal development and differentiation of progenitor cells may provide valuable insight into alternative approaches to increase the yield of specific retinal cell subpopulations. Optimizing the yield of certain subtypes will have direct implications for future cell replacement strategies for degenerative retinal diseases.

Deleted in Breast Cancer 1 (DBC1), initially cloned from a homozygously deleted region (8p21) in breast cancers, was shown to be an innate inhibitor of SIRT1 by two independent studies in 2008 [16, 17]. The studies suggested a mechanism whereby DBC1 directly interacts with SIRT1 and inhibits its activity. Thus, evaluating the expression of DBC1 alongside SIRT1 may provide additional insight into SIRT1 biology. To date, DBC1 expression and localization have not been investigated in the eye.

The purpose of this study was to evaluate the expression of SIRT1 and its innate inhibitor, DBC1, in human and mouse retinal tissues and cells in an effort to elucidate a possible role for these proteins in governing retinal development.

## 2. Results

### 2.1. SIRT1 and DBC1 Expression in Human and Mouse Eyes

**Figure 1:**
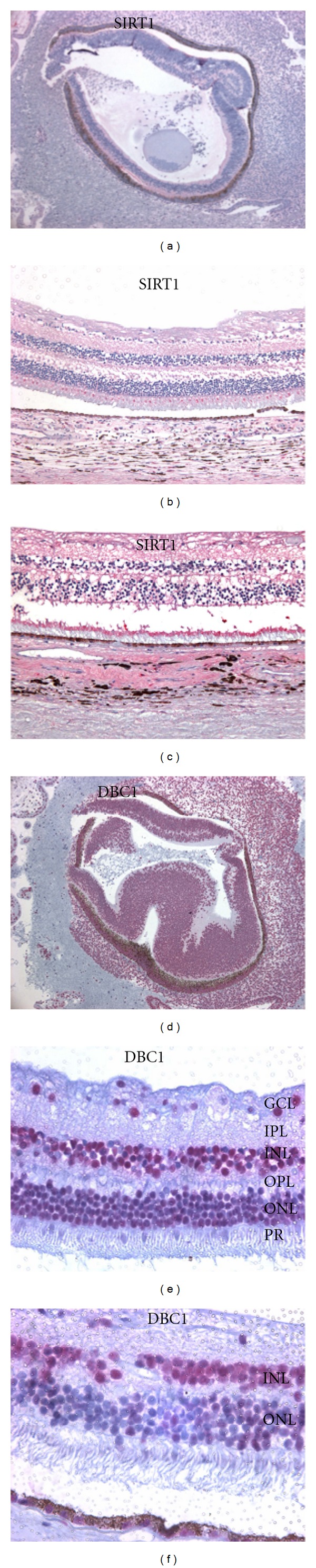
Expression of SIRT1 and DBC1 in fetal and adult human eyes. SIRT1 was exclusively cytoplasmic in the retinas of all fetal ((a): 100x) and adult ((b)-(c): 160x) eyes and seen in GCL, IPL, OPL, and photoreceptor (PR) inner segments. DBC1 was exclusively nuclear in fetal eyes ((d): 100x) with stronger staining in the primitive INL than the ONL. In adult eyes ((e): 400x, (f): 640x), DBC1 was exclusively nuclear in the INL and ONL and both nuclear and cytoplasmic (weak) in the GCL. Of note, GCL and INL nuclei stained stronger than ONL nuclei in all specimens.

To evaluate SIRT1 expression in human retinas, we immunostained eye sections from human donors of various ages. Additionally, we examined the expression of the innate SIRT1 inhibitor, DBC1, in the same specimens. The immunohistochemistry results demonstrated that SIRT1 was exclusively cytoplasmic in all human retinas studied (Figures 1(a)–1(c)) and DBC1 was exclusively nuclear, with the exception of the ganglion cell layer (GCL) where it was both nuclear and cytoplasmic (Figures 1(d)–1(f)). Importantly, all retinal specimens stained positive for SIRT1 in the GCL, inner plexiform layer (IPL), outer plexiform layer (OPL), and photoreceptor inner segments, while all specimens were DBC1 positive in the GCL, inner nuclear layer (INL), and outer nuclear layer (ONL), with the GCL and INL showing stronger expression than the ONL in all cases.

**Figure 2:**
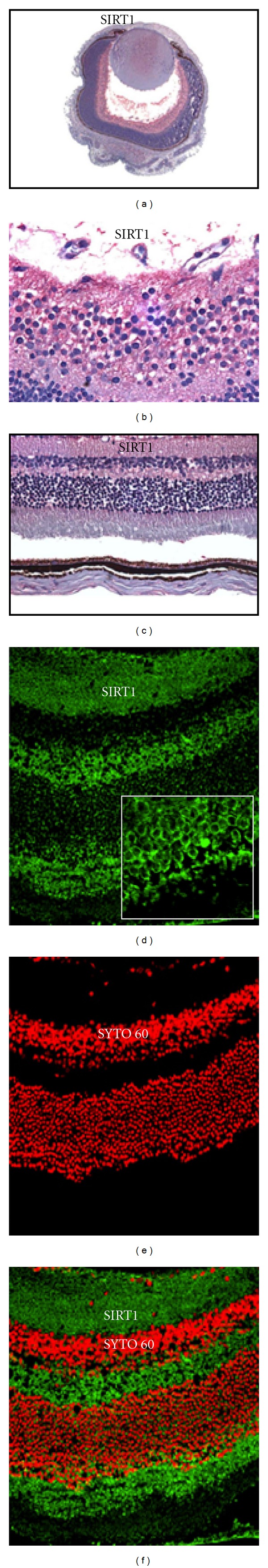
SIRT1 expression in mouse eyes. (a) Postnatal day 1 (P1) mouse eye (50x). (b) Higher magnification of (a) (640x). Ganglion cells and nerve fibre layer stained positive. (c) Inner and outer plexiform layers stained positive while the inner and outer nuclear layers are negative for SIRT1 in this specimen (P244 mouse eye; 400x). (d) SIRT1 staining in adult mouse retina (green). Insert highlights cytoplasmic staining in ONL. (e) SYTO 60 nuclear stain of retinal cell nuclei (red). (f) Composite image of (d) and (e) showing predominant cytoplasmic localization of SIRT1 in this section.

We next sought to determine whether or not SIRT1 and DBC1 were expressed in mouse retinas. Accordingly, we immunostained eyes from mice of various ages to see if the staining pattern was the same as in human retinas. For SIRT1, strong cytoplasmic staining was seen in the IPL, OPL and photoreceptor inner segments in all eyes (Figures 2(a)–2(f)). The ONL was consistently negative while sparse positivity was seen in the INL in some eyes (*n* = 8). The GCL was consistently positive in all eyes and cytoplasmic, with two eyes showing very weak nuclear staining in addition to cytoplasmic positivity. No statistically significant correlations existed between nuclear localization and age. Notably, P1 eyes had cytoplasmic staining only (Figures 2(a)-2(b)).

DBC1 was strongly expressed in the GCL nuclei and INL, while moderately expressed in the ONL in all eyes. This pattern was consistent in all eyes, including P1 mouse eyes. Of note, this was also the same pattern seen for DBC1 in human eyes. Collectively, these results suggest that SIRT1 and DBC1 are expressed in human and mouse retinas and that the patterns of expression are similar with some subtle differences.

### 2.2. SIRT1 Expression in Mouse Retinal Progenitor Cells (mRPCs)

**Figure 3:**
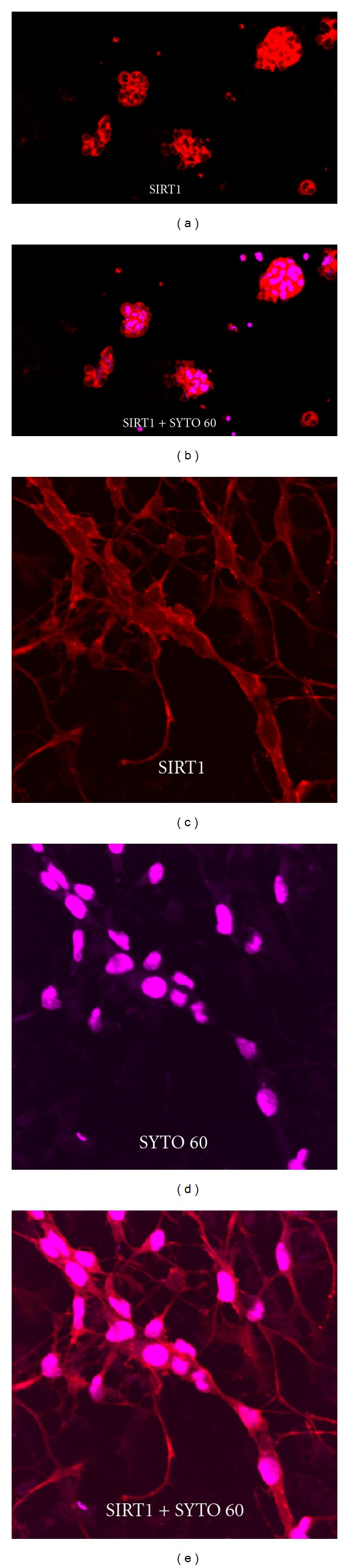
Expression of SIRT1 in mouse retinal progenitor cells. (a) SIRT1 positivity (red) seen throughout neurosphere. (b) Composite image showing SIRT1 staining (red) and SYTO 60 nuclear staining (magenta). (c) Expression of SIRT1 (red) in differentiated mRPCs. (d) SYTO 60 nuclear stain (magenta). (e) Composite of (c) and (d) showing cytoplasmic expression of SIRT1 in differentiated mRPCs.

Immunohistochemistry of mouse eyes demonstrated widespread expression of SIRT1 throughout the retina at all ages. Thus, we sought to determine whether or not SIRT1 was expressed in undifferentiated and differentiated mouse retinal progenitor cells. Confocal microscopy of immunostained cytospins of mRPCs revealed ubiquitous expression of SIRT1 in mRPC neurospheres (Figures 3(a)-3(b)). After differentiating cells in culture for 7 days, SIRT1 expression was evaluated and seen to be predominantly cytoplasmic (Figures 3(c)–3(e)).

Previous *in vitro* studies with neural progenitor cells observed a transient shuttling of SIRT1 during differentiation from the cytoplasm to the nucleus and subsequently back to the cytoplasm [14]. Thus, to determine if the same transient shuttling occurs in mRPCs, we evaluated SIRT1 subcellular localization at different time points after transferring cells into differentiating media. Our results revealed that nuclear shuttling of SIRT1 did not occur; it was expressed in the cytoplasm exclusively at all time points assayed (data not shown). This result was consistent for cells from both passage 9 and passage 17, suggesting that the shuttling observed in neural progenitors may not occur in retinal progenitors of mouse origin.

### 2.3. SIRT1 and DBC1 Subcellular Localization in Human Retinal Progenitor Cells (hRPCs)

While no nuclear shuttling of SIRT1 was seen in mRPCs, we wanted to further test this possibility using hRPCs, particularly because some differences in both SIRT1 and DBC1 expression were seen in mouse and human retinas.

**Figure 4:**
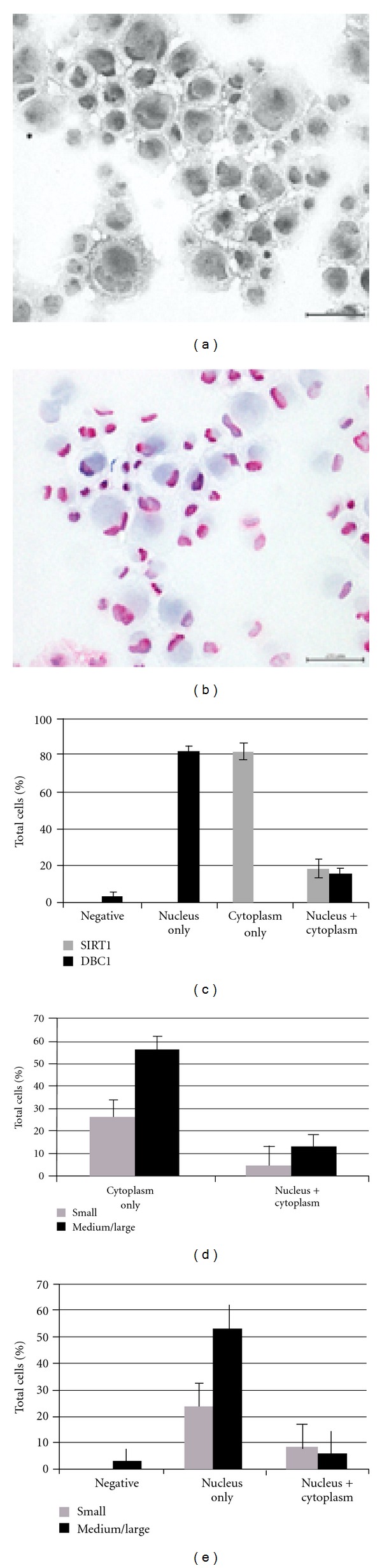
(a) Light microscope image of hRPCs immunostained for SIRT1. Immunoreactivity can be seen predominantly in the cytoplasm but also in the nuclei of some cells. (b) hRPCs immunostained for DBC1. Immunoreactivity can be seen predominantly in the nuclei but also in the cytoplasm of some cells. (c) Distribution of SIRT1 and DBC1 staining in hRPCs. (d) SIRT1 staining classification and hRPC cell size. Of note, SIRT1 positivity was observed in both the cytoplasm and nucleus in hRPCs of all sizes. No significant difference in SIRT1 distribution was observed for small versus medium/large cells. (e) DBC1 staining classification and hRPC cell size. Of note, DBC1 positivity in the cytoplasm was more frequently seen in small cells compared to medium/large cells. Also, some medium/large cells were negative for DBC1 whereas all small cells were DBC1 positive.

The first observation we made about these cells was that they were a heterogeneous population with respect to cell size, similar to what was seen for mRPCs. Thus, in characterizing SIRT1 immunostaining, we also considered the size of the cells. Relative size was considered rather than absolute size in all cases. Results demonstrated that SIRT1 expression could be detected in both the nucleus and cytoplasm of hRPCs as a population (Figure 4(a)). The distribution of staining is summarized in Figures 4(c)-4(d).

Based on the observation that SIRT1 was localized in the nuclei of some cells, we chose to evaluate the expression of DBC1 in the same manner to further clarify SIRT1's possible role in retinal development. Like SIRT1, DBC1 was also found to be localized to both the nucleus and cytoplasm in some cells (Figures 4(b)-4(c), and 4(e)).

## 3. Discussion

SIRT1 has emerged as a critical regulator of many physiological and pathological processes. Interest has also grown in DBC1 as a negative regulator of SIRT1, particularly in the cancer literature [18, 19]. To date, little is known about the roles of SIRT1 and DBC1 in retinal physiology. We found both proteins to be widely expressed throughout mouse and human retinas at various ages, including during development.

In all eyes studied, SIRT1 was predominantly cytoplasmic, although some nuclear localization was seen in a subset of mouse retinas. While the function of SIRT1 in cell nuclei has been well described for many cell types, its function in the cytoplasm is less clear. Nuclear SIRT1 is known to be associated with deacetylation of transcription factors that can regulate cell cycle, apoptosis, and other processes. It is possible that subcellular localization of SIRT1 in the retina is dynamic and that transient nuclear localization occurs when the retina experiences stress or during development. A high basal level of cytoplasmic SIRT1 would allow for nuclear shuttling and a rapid SIRT1-mediated response in times of cellular stress. The notion that stress and other physiological conditions can influence subcellular localization of SIRT1 has been reported elsewhere [15].

Tanno et al. evaluated the expression of SIRT1 in mouse tissues and found it to be exclusively cytoplasmic, exclusively nuclear, or both cytoplasmic and nuclear in various tissues [20]. Notably, some subsets of neurons predominantly expressed SIRT1 in the cytoplasm, a finding similar to what we found in the retina. While no such analysis has been done for human tissues, it appears that the subcellular localization of SIRT1 may differ across tissue types and even across cell types within a specific tissue. Moreover, our findings suggest that subtle differences may exist across species with respect to subcellular distribution of SIRT1 given that some mouse eyes studied exhibited nuclear expression of this protein. However, other variables such as environmental stress cannot be ruled out as causes of this nuclear localization.

Aside from some cytoplasmic expression of DBC1 in the GCL in human specimens, DBC1 staining was remarkably similar in mouse and human retinas. A distinct pattern whereby the GCL and INL stained stronger than the ONL in all specimens was evident. DBC1 is known to interact with many proteins, including SIRT1, estrogen receptor alpha (ER*α*), RAR*α*, and others [21, 22]. It is also a protein of interest in some cancers, where its expression has been shown to be deregulated compared to control tissues [18, 23]. Additionally, DBC1 has been shown to be a key mediator of apoptosis and TNF-*α* signalling [24]. To date, the role of DBC1 in neural tissue is attributed primarily to its function as an SIRT1 inhibitor. The nuclear localization of DBC1 that we observed in human retinas may be important for inhibiting SIRT1-mediated deacetylation of nuclear proteins such as transcription factors. Although SIRT1 expression was cytoplasmic, environmental cues may cause its nuclear shuttling. Whether or not DBC1 remains active and nuclear in times of stress or development, and thus inhibits SIRT1 activity, remains to be determined. The stronger expression of DBC1 consistently observed in the GCL and INL compared to the ONL in all mouse and human retinas was a particularly interesting observation. One study previously found that bovine and rat retinas express estrogen receptor to a greater extent in the GCL and INL compared with the ONL [25]. Given that DBC1 can inhibit estrogen receptor, the similar patterns of staining may be of significance, although a more comprehensive evaluation of DBC1 function is necessary before any conclusions can be drawn.

The cytoplasmic expression of DBC1 in ganglion cells is also worth noting. The majority of studies that have looked at DBC1 have referred to it as a nuclear protein. Caspase-dependent cleavage of DBC1 can cause its cytoplasmic localization where it functions to promote apoptosis [24]. It is unlikely that the role of cytoplasmic DBC1 in normal human ganglion cells is promotion of apoptosis, unless there are other mediators keeping this activity in check. Cytoplasmic DBC1 in these cells may also regulate cytoplasmic SIRT1 function; this hypothesis is currently being investigated by our group.

Evidence from studies with neural progenitor cells suggests that SIRT1 may be important in the differentiation of this cell type and that its role may be dependent upon its translocation to the nucleus where it can deacetylate transcription factors that influence cell fate determination. Accordingly, we investigated the expression and distribution of SIRT1 in mouse retinal progenitor cells before and after differentiation at various time points. While SIRT1 was ubiquitously expressed, the absence of its nuclear localization in every experimental condition suggests that either shuttling of SIRT1 does not occur in this cell type or that this process could not be reproduced *in vitro* in the same manner as it was for neural progenitor cells. This may be due to cell-specific or differentiation protocol-specific differences.

The study by Hisahara and colleagues showed that SIRT1 shuttling to the nucleus was critical for the differentiation of neural progenitors into mature neurons [14]. Accordingly, we expected to see some fetal retinal cells expressing nuclear SIRT1. It is possible that nuclear shuttling of SIRT1 does contribute to retinogenesis but that this shuttling occurs at an earlier or later time point in gestation than what was evaluated in our study (approximately 10 weeks). It is also conceivable that such nuclear shuttling occurs at various points during retinogenesis and in specific subpopulations of retinal progenitors. In our analyses, we saw SIRT1 nuclear localization in some hRPCs. These cells were derived from 14-week-old fetuses, supporting the idea that SIRT1 nuclear shuttling happens later than 10 weeks of gestation. This was also confirmation that nuclear shuttling of SIRT1 in human retinal cells does occur during development given that SIRT1 was exclusively cytoplasmic in adult human retinas. Further work will need to be done with a large series of fetal retinas before correlations between gestational age and nuclear shuttling of SIRT1 can be established.

The identification of nuclear SIRT1 in some hRPCs was the impetus to evaluate DBC1 expression in this cell type. As expected, DBC1 was predominantly nuclear, although coexpression in the cytoplasm was observed in some cells (Figure 4). Interestingly, there was a notable difference in the distribution profile for DBC1 in small and medium/large cells whereby cytoplasmic expression was more frequently seen in small cells. The significance of this observation lies in whether or not cells of different sizes are lineage specific, which is currently unclear. However, in adult eyes we did observe cytoplasmic localization of DBC1 in the GCL, suggesting that hRPCs with cytoplasmic DBC1 may potentially be ganglion cell precursors, although extensive analyses are required before this hypothesis can be confirmed. The fact that we observed DBC1-negative cells suggests that DBC1 expression may be dynamically regulated during retinal development given that it was found to be ubiquitously expressed in the GCL, INL, and ONL of adult retinas.

We found SIRT1 to be ubiquitously expressed in retinal progenitor cells which suggests that it may be important in retinal development. Its nuclear localization in some hRPCs further highlights that its subcellular distribution is dynamic and may contribute to the genesis of specific retinal cell subtypes. Widespread expression of SIRT1 in adult retinas additionally suggests that it may have a role in normal retinal physiology, possibly serving as a redox sensor that shuttles to the nucleus during times of environmental stress to enable a rapid cellular response. The sparse nuclear-localized SIRT1 seen in the INL and GCL of some mouse eyes may be the result of an unidentified stress or possibly due to subtle species-specific differences. DBC1, a potent negative regulator of SIRT1, was found to be expressed throughout mouse and human retinas and was exclusively nuclear except for its coexpression in the cytoplasm of human ganglion cells. Cytoplasmic DBC1 was also seen in a subset of hRPCs, indicating that this subset, comprised predominantly of small cells, may represent a population of ganglion cell precursors. Collectively, the data presented in this study provide evidence of SIRT1 and DBC1 expression in developing and adult retinas. Further functional analyses are needed in order to elucidate definitive roles for both proteins in the developing and adult retina.

## 4. Experimental Procedures

### 4.1. Tissue Collection

#### 4.1.1. Mouse Eyes

All mouse eyes used were generously donated by the laboratory of Dr. Michael Young (Schepens Eye Research Institute, Harvard University, Boston, MA). A total of 24 mouse eyes from C57bl6 mice ranging in age from postnatal day 1 (P1) to P347 were used. P1 mouse eyes were used to study the developing retina as mouse retinas are not fully mature at this stage (Dorrell et al., 2004). All eyes were put in 10% formalin for 48 hours prior to processing and paraffin embedding.

#### 4.1.2. Human Eyes

Human donor eyes were obtained from the Eye Bank of Canada (Toronto, ON) and received in 10% formalin and processed and embedded upon receipt. A total of nine eyes from five human donors were used for immunohistochemical analysis. All eyes met the inclusion criteria established for donor control eyes, namely, no reported ocular disease upon fundoscopic examination and no death due to head trauma or sepsis. Tissues from 3 female donors (aged 17, 53, and 90 years) and 2 male donors (aged 58 and 87 years) were used. In addition, sections from two human fetal eyes (approximately 10 weeks of gestation) were obtained by request for immunohistochemical studies. Fetal eyes were not subjected to the same inclusion criteria as adult eyes given that history of these tissues was unknown.

### 4.2. Tissue Preparation

All human and mouse eyes were processed following formalin fixation. Human eyes were dissected in a transverse plane from cornea to optic nerve, ensuring that the cut went through the macula. The two separate halves of each globe were put into tissue cassettes and sent for paraffin embedding. Mouse eyes were sent for paraffin embedding without first dissecting the eyes due to the size of the eyes and the risk of disrupting the natural intraocular architecture. Four-micrometer-thick sections of paraffin-embedded eyes were cut and placed on glass microscope slides. Multiple sections were cut from each embedded eye for immunohistochemical studies.

### 4.3. Immunohistochemistry

Antibodies against SIRT1 (Abcam) and DBC1 (Abcam) were used to stain both human and mouse tissues. All immunohistochemistry was done using the Ventana BenchMark fully automated system. The processing of barcode-labelled slides included baking of the slides, solvent-free deparaffinization, and CC1 (Tris/EDTA buffer, pH 8.0) antigen retrieval. Slides were incubated with SIRT1 or DBC1 antibodies for 30 minutes at 37°C, followed by application of a biotinylated secondary antibody (8 minutes at 37°C) and an avidin-alkaline phosphatase conjugate complex (8 minutes at 37°C). Finally, the antibody was detected by Fast Red chromogenic substrate and counterstained with hematoxylin. As positive controls, sections of brain and colon cancer were used for SIRT1 and DBC1, respectively. For negative controls the primary antibody was omitted. All human and mouse eyes were examined for immunopositivity in the neural retina.

Images of immunostained tissues and cells were taken using a Zeiss Axiophot microscope equipped with a Zeiss AxioCam digital camera. Immunofluorescence images were taken using an Olympus FluoView FV1000 Confocal Microscope. Images were cropped and assembled into image composites using Adobe Photoshop CS5 software.

### 4.4. Mouse Retinal Progenitor Cells (mRPCs)

Retinal progenitor cells harvested from the eyes of postnatal day 1 GFP-transgenic mice were generously provided by the laboratory of Dr. Michael Young. GFP expression was not evaluated for any purpose in the current study. Cells isolated from P1 mouse eyes were cultured in 25 cm^2^ flasks in Neurobasal media containing 2 mM L-glutamine, 100 mg/mL penicillin streptomycin, 20 ng/mL epidermal growth factor (EGF), 1 ug/mL fungizone, and N2 and B27 neural supplements (Invitrogen-Gibco). Cells were passaged 1 : 3 every 7–10 days. For studies involving differentiation of mRPCs, growth factor-free media was supplemented with 5% fetal bovine serum (FBS). Cells were cultured with this media for 7 days to induce differentiation, with half of the media being changed every 48 hours.

### 4.5. Immunofluorescence

For immunofluorescence studies, mouse retinal progenitor cells were seeded in 8-well chamber slides (LabTech) and left for a minimum of 24 hours before immunofluorescence staining. SIRT1 expression was investigated in mRPCs that were either undifferentiated or differentiated for 7 days. A Cy3 secondary antibody was used, and analysis and imaging were performed using confocal microscopy to evaluate SIRT1 expression and subcellular distribution.

### 4.6. mRPC Differentiation: Time Course Analysis

Passage 9 and 17 mRPCs were used to assess the subcellular localization of SIRT1 protein before and immediately after moving cells into differentiating media. Briefly, mRPCs were seeded in a 12-well plate in modified Neurobasal media and left overnight. The next day, FBS was added at specific time intervals to individual wells to a final concentration of 5% by volume. The following conditions were set up: control (undifferentiated) and 1, 3, and 5 hours in differentiating media. The same conditions were set up for passage 9 and passage 17 cells. Cells were all harvested at the same time and immediately used to make cytospins, which were then immunostained using an SIRT1 antibody (Abcam). Immunocytochemistry was performed using the Ventana Benchmark system as described in Section 4.3. Immunostained cytospins were then evaluated microscopically to discern the subcellular localization of SIRT1 at each experimental time point.

### 4.7. Human Retinal Progenitor Cells (hRPCs): Culture and Immunocytochemistry

Human retinal progenitor cells (hRPCs) were generously provided by the laboratory of Dr. Michael Young. Cells were derived from human fetuses (14 weeks' gestational age) that were obtained from Advanced Bioscience Resources (Alameda, CA) through therapeutic termination of pregnancy. Cells from passage 3 were used in our studies.

Cells were cultured at 37°C and 5% CO_2_ in Ultraculture media (Lonza) supplemented with 2 mM L-glutamine, 10 ng/mL epidermal growth factor (EGF), 20 ng/mL basic fibroblast growth factor (bFGF), penicillin-streptomycin (100 mg/mL), and fungizone (1 ug/mL). Cells were cultured in 25 cm^2^ flasks and fed every 2-3 days, which involved removing half of the media from the flask and replacing it with an equivalent volume of fresh media. Cells were grown to approximately 70–80% confluency at which point they were either harvested for experiments or passaged.

Cytospins of hRPCs were made, and immunocytochemistry was performed using the Ventana Benchmark system as described in Section 4.3. Separate slides were stained using SIRT1 and DBC1 antibodies. Subsequent evaluation of expression and subcellular localization of each stained marker was achieved via 10 high power field counts (400x) per slide under a standard light microscope.
